# State Benefit Issuance Systems and Prenatal WIC Participation Trends During the COVID-19 Pandemic

**DOI:** 10.3390/nu18213484

**Published:** 2026-10-23

**Authors:** Janay R. Johnson, Leslie Hodges, Marie E. Thoma

**Affiliations:** 1 Department of Family Science, School of Public Health, University of Maryland, 4200 Valley Drive, Family Science Suite 1142, College Park, MD 20742, USA; janayj@umd.edu; 2 Economic Research Service, U.S. Department of Agriculture , 805 Pennsylvania Avenue, Kansas City, MO 64105, USA; leslie.hodges@usda.gov

**Keywords:** WIC, prenatal, COVID-19 pandemic, pregnancy, service delivery, maternal, infant

## Abstract

**Background:** Prenatal participation in the U.S. Department of Agriculture’s Special Supplemental Nutrition Program for Women, Infants, and Children (WIC) improves maternal and infant health. To reduce administrative burden in WIC benefit redemption, the Healthy, Hunger-Free Kids Act required states to adopt electronic benefit transfer (EBT) systems by October 2020; however, the COVID-19 pandemic created a major disruption to this implementation and WIC service delivery. This study examines the impact of state WIC benefit issuance systems on prenatal WIC participation at the time of birth during the COVID-19 pandemic. **Methods:** We applied a quasi-experimental difference-in-differences approach using U.S. birth certificate data linked to state economic indicators (January 2019–December 2021). The sample included all Medicaid births (*n* = 4,564,705). States were classified by the type of benefit issuance adopted as of March 2020: online EBT, offline EBT, transitioning to an EBT system, and paper vouchers. Linear probability models were fit with interaction terms between each benefit issuance group and time (pre-pandemic and pandemic 2020 or 2021). Models adjusted for maternal and infant characteristics and state-level economic indicators. **Results:** Prenatal WIC participation declined during the pandemic in all benefit issuance groups. States transitioning to an EBT system (β = −2.76, 95% CI: −4.61, −0.91 in 2021) or remaining on paper vouchers (β = −3.17, 95% CI: −5.47, −0.87 in 2021) experienced the largest pandemic-related declines compared with online EBT states. We found no significant difference between offline and online EBT groups. **Discussion:** Adoption of either online or offline EBT systems mitigated WIC participation declines during the pandemic. These findings have implications for ongoing WIC service delivery modernization efforts to safeguard prenatal WIC access from future external shocks.

## 1. Introduction

Adequate prenatal nutrition is essential to the health of both the pregnant woman and her infant(s) [1,2]. The U.S. Department of Agriculture’s (USDA) Special Supplemental Nutrition Program for Women, Infants, and Children (WIC) was established in 1972 to support the health of low-income, nutritionally at-risk pregnant and postpartum women and children less than 5 years old [3]. WIC provides nutritious supplemental foods, education, breastfeeding support, and referrals to healthcare and social service programs. Several studies have shown that WIC participation is associated with improved health outcomes for women and children, including greater intake of nutrient-dense foods, increased health care access, better birth outcomes, and enhanced child cognitive development [2,4,5,6]. These health benefits are maximized when participants are able to enroll and receive services early in the prenatal period. Until recently, WIC participation had been in a decade-long decline since 2010 [7]. Some of this decline has been attributed to reductions in food insecurity and birth rates after the 2008 recession [8]; however, recent estimates indicate only half of all income-eligible pregnant women participate in WIC (49.3 in 2023) [9].

Several studies demonstrate programmatic and administrative burdens can further deter WIC-eligible individuals from enrolling in WIC [10,11]. Prior to 2010, U.S. states used paper vouchers to redeem WIC food benefits, which were shown to diminish the user experience and increase stigma [12,13,14]. Recognizing the need for system-level changes to reduce participant burden, the U.S. Congress passed the Healthy Hunger-Free Kids Act in 2010 that required all states to transition from the use of paper vouchers to electronic benefit transfer (EBT) debit card systems by 1 October 2020 [15]. The goal was to streamline the redemption process for vendors and ensure a flexible and efficient shopping experience for the WIC beneficiary through enhanced card technology. States adopted one of two methods for implementing EBT systems consistent with state regulations and information system capacity: (1) offline EBT in which food benefit data is embedded onto a “smart card” and reloaded every 3–4 months via mail or in person, or (2) online EBT in which benefits are reloaded remotely every month [16,17,18]. Although online EBT systems allow for remote reloading of benefits, certification and issuance of EBT cards or paper vouchers remain in person [19]. Prior to the October 2020 deadline, the onset of the COVID-19 pandemic created an abrupt “shock” that disrupted EBT modernization efforts, redirected health department resources, and strained ongoing WIC benefit issuance and delivery [19].

The emergence of the COVID-19 pandemic in March 2020 upended previously declining U.S. food insecurity trends, and an economic recession ensued [20,21,22]. Not only were resources constrained due to increased job losses or reduced work hours, but health and social service access and delivery were disrupted [23,24]. In response, the Families First Coronavirus Response Act of 2020 (FFCRA, P.L. 116–127) was enacted, which allowed the USDA to adapt food assistance programs to ensure continued access to WIC services [25]. Between March 2020 and February 2021, the USDA issued waivers for state WIC agencies to adhere to COVID-19 social distancing precautions by relaxing in-person requirements for remote certification and delivery of WIC services. USDA reports document that nearly all (98.6%) local WIC agencies adopted the waiver and conducted certifications remotely, with telephone being reported as the most common method used (98.5%) [19]. During this time, overall WIC enrollment increased by 2% with considerable variability by state (range: 21% decline in Arkansas, 18% increase in California) [26]. In contrast, WIC enrollment patterns for pregnant and postpartum individuals showed a 5% decline and wide variation by state during this same period (range: 20% decline in Arkansas, 7% increase in New Hampshire) [26].

One study found that increases in WIC participation were concentrated in states with online EBT systems while states with offline EBT systems declined following the start of the COVID-19 pandemic [16]. They attributed these differences to greater reliance on in-person and mail delivery for issuing benefits by states with offline EBT systems, which acted as a disincentive to enrollment. A similar study examining state WIC policy changes during the COVID-19 pandemic also found states with online EBT systems had higher participation rates for both women and children [27]. However, neither of these studies examined participation among pregnant women—a population that may benefit most from early WIC enrollment.

Given pandemic-related differences in overall WIC enrollment patterns (increases) compared to patterns among pregnant and postpartum women (declines) and the importance of prenatal WIC enrollment for optimizing maternal and child health outcomes, it may be informative for policymakers and program staff to understand whether benefit issuance program design features operate similarly in the prenatal period. Therefore, the objective of this study was to examine whether state benefit issuance systems exhibited differential patterns of prenatal WIC participation before and during the COVID-19 pandemic. These findings can guide policy efforts aimed at strengthening the resilience of WIC benefit issuance systems and promote timely WIC enrollment during periods of program disruption or public health emergencies.

## 2. Materials & Methods

### 2.1. Data Source and Study Population

We analyzed national birth certificate data with state and county geographic identifiers from 2019 to 2021, which represent 100% of all registered live births occurring in the United States each year [28]. These de-identified data were obtained from the National Center for Health Statistics, Centers for Disease Control and Prevention. We merged birth certificate data with other state-level characteristics using Federal Information Processing Standard (FIPS) identifiers for each state. State-specific data for 2019 through 2021 included annual median household income and percent below the poverty level, obtained from annual data tables derived from the American Community Survey (2019–2021) and monthly unemployment rates from the Bureau of Labor Statistics Local Area Unemployment Statistics (LAUS) [29,30]. The University of Maryland, College Park Institutional Review Board determined this study was not human subjects research. We applied the Strengthening the Reporting of Observational Studies in Epidemiology (STROBE) guidelines.

The analytic sample included all births in which the delivery was paid for by Medicaid and the birthing person was a U.S. resident (*n* = 4,564,705 births). We restricted to Medicaid-only births to ensure our sample was eligible for WIC services via adjunctive eligibility (i.e., being eligible to enroll in WIC because they are eligible for another federal assistance program, such as Medicaid) [31].

### 2.2. Study Measures

We determined WIC participation from birth certificates based on the mother’s response to the question “Did you receive WIC (Women, Infants & Children) food for yourself because you were pregnant with this child?” This measure has been reported with high reliability compared with Pregnancy Risk Assessment Monitoring System data (*k* = 0.81) [32]. We used month and year of birth to define the time periods under study. First, we reviewed monthly trends in WIC participation to ensure our time periods aligned with descriptive patterns. Additionally, we considered the changing context of the COVID-19 pandemic (i.e., vaccine availability and new SARS-CoV-2 variants in 2021). Additionally, births occurring in April 2020 or before were assumed to reflect enrollment experiences prior to the start of the pandemic. As such, we partitioned the month and year of births into 3 categories relative to the start of the COVID-19 pandemic: pre-pandemic (January 2019–April 2020), pandemic 2020 (May 2020–December 2020), and pandemic 2021 (January 2021–December 2021).

**Figure 1:**
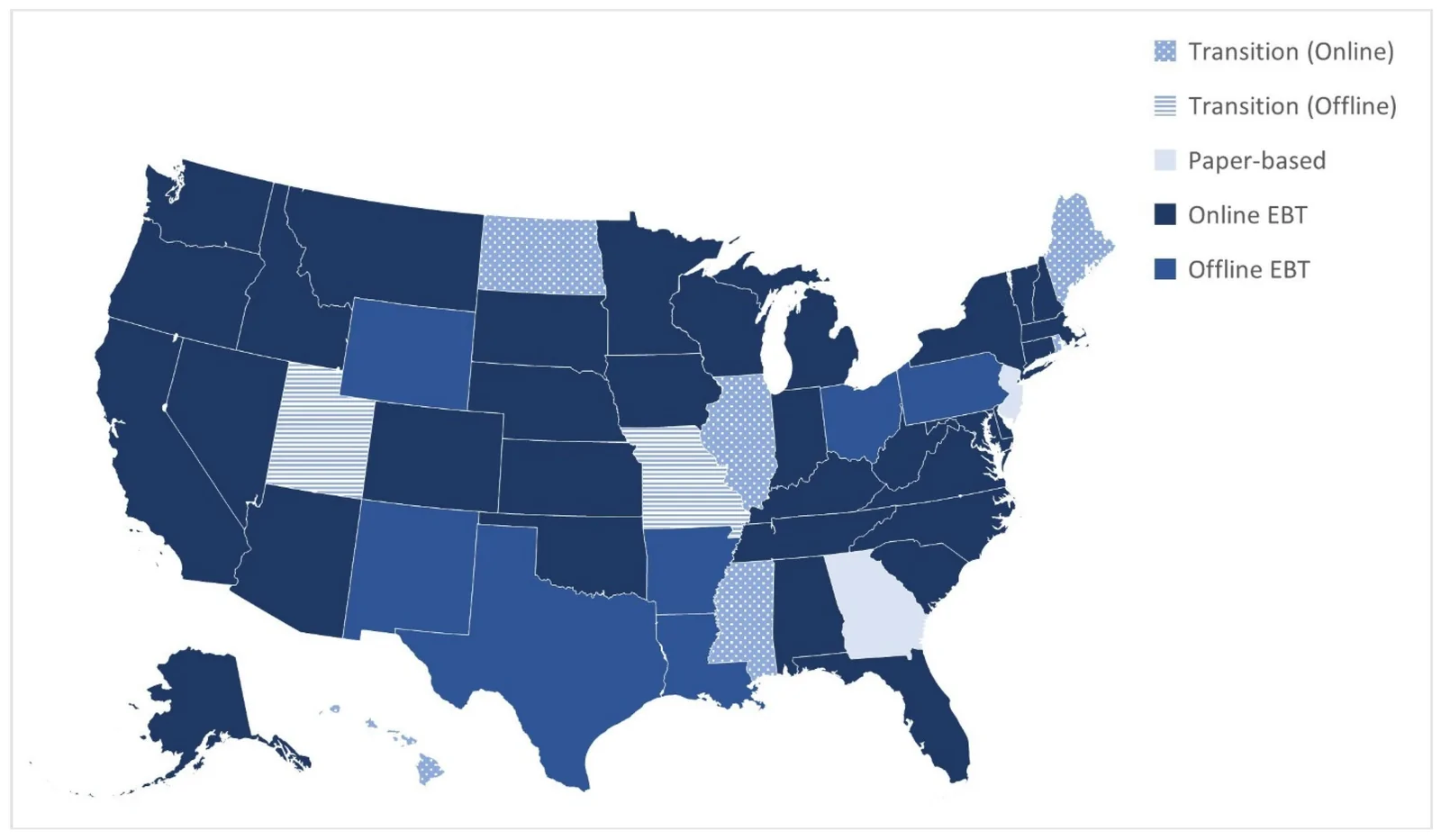
Status of state benefit issuance systems as of March 2020.

The USDA WIC EBT Detail Status Report provided information on state WIC agency benefit issuance status, which we classified based on whether or not a state WIC agency had initiated adoption of an online or offline EBT system by the start of the COVID-19 pandemic in March 2020 (Figure 1) [33]. As of March 2020, states operated under one of five WIC benefit issuance systems: (1) an offline EBT system that required WIC beneficiaries to mail or bring their EBT cards to an office in person to reload their benefits every 3 to 4 months, (2) an online EBT system that allowed WIC beneficiaries to automatically reload EBT benefits on their cards remotely every month, (3) paper vouchers that were received in person every month, (4) state systems that were in the process of transitioning from paper vouchers to online EBT systems, and (5) state systems that were in the process of transitioning from paper vouchers to offline EBT systems [17].

Thirty-three states initiated online EBT systems prior to the pandemic (Alaska, Alabama, Arizona, California, Colorado, Connecticut, Delaware, Florida, Iowa, Idaho, Indiana, Kansas, Kentucky, Massachusetts, Maryland, Michigan, Minnesota, Montana, North Carolina, Nebraska, New Hampshire, Nevada, New York, Oklahoma, Oregon, South Carolina, South Dakota, Tennessee, Virginia, Vermont, Washington, West Virginia, and Wisconsin). Seven states (Arkansas, Louisiana, New Mexico, Ohio, Pennsylvania, Texas, and Wyoming) had initiated offline EBT systems prior to the pandemic. Six states and the District of Columbia transitioned from paper vouchers to an online EBT system during the pandemic (Hawaii, Illinois, Maine, Mississippi, North Dakota, and Rhode Island), while two states transitioned from paper vouchers to offline systems (Utah and Missouri). Finally, two states remained on paper-based benefit issuance systems throughout the pandemic (Georgia and New Jersey).

We examined several maternal demographic and health, infant health, and state-level characteristics as covariates in this study. Maternal demographic characteristics included age, marital status, education level, and racial/ethnic group. Maternal health characteristics included parity, body mass index (BMI), diabetes, hypertension, and timing of prenatal care initiation. Infant health characteristics included whether the infant was born preterm (i.e., less than 37 weeks gestation) or low birthweight (i.e., less than 2500 g). Lastly, state-level characteristics included the mean poverty rate and household income per year and mean unemployment rates per month.

### 2.3. Statistical Analyses

We applied a difference-in-differences approach to estimate the average difference in the probability of prenatal WIC participation at birth by state benefit issuance system and timing of birth. We fit linear probability models (LPMs) with interaction terms between state benefit issuance groups and pre- and pandemic time periods and accounted for clustering of individuals within states using a cluster-robust (sandwich) variance estimator. LPM provides an alternative and less computationally intensive approach for obtaining average marginal effects, particularly when the sample size is large [34].

We produced unadjusted and adjusted models that accounted for maternal sociodemographic (age, education, race/ethnicity), maternal health (parity, BMI, smoking, hypertension, diabetes, and prenatal care initiation), and state-level characteristics (mean poverty rate, monthly unemployment rate, and median household income) using complete-case analysis. Marital status was included in the demographic table but was not controlled for in the regression models because NCHS stopped releasing record-level data on the marital status of mothers in California due to state statutory restrictions in 2017. Initial descriptive trends and models examined all 5 state benefit issuance groups. WIC participation patterns in states transitioning to an EBT system, either online or offline, were similar descriptively and not significantly different in adjusted models (*p* = 0.73 and 0.81 for interaction terms for states that transitioned to online compared to offline in pandemic periods of 2020 and 2021, respectively). Therefore, we combined these groups together for a total of four benefit issuance groups in our main analyses: online EBT, offline EBT, transition to an EBT system, and paper vouchers. Adjusted percentages of prenatal WIC participation before and during the pandemic were plotted by benefit issuance group from fully adjusted LPMs. The statistical software used for data analysis was STATA version 19.5 (College Station, TX, USA).

## 3. Results

The distributions of maternal demographic and health, infant health, and state-level characteristics by birth year and benefit issuance group were examined in Table 1. Trends in demographic characteristics over time were relatively similar across each group. Overall, we found a decline in births among mothers <20, married, with less than high school education, and smoking before and during pregnancy. There were also increases in births among mothers with higher BMI categories (overweight and obese), diabetes, hypertension, receipt of prenatal care in the first trimester, and infants born preterm or low birthweight between 2019 and 2021. For state characteristics, the poverty rate increased in 2020 and remained stable in 2021, whereas the unemployment rate increased in 2020 and decreased in 2021. Consistent with this trend, median household income decreased in 2020 but increased again in 2021 to rates above 2019.

**Figure 2:**
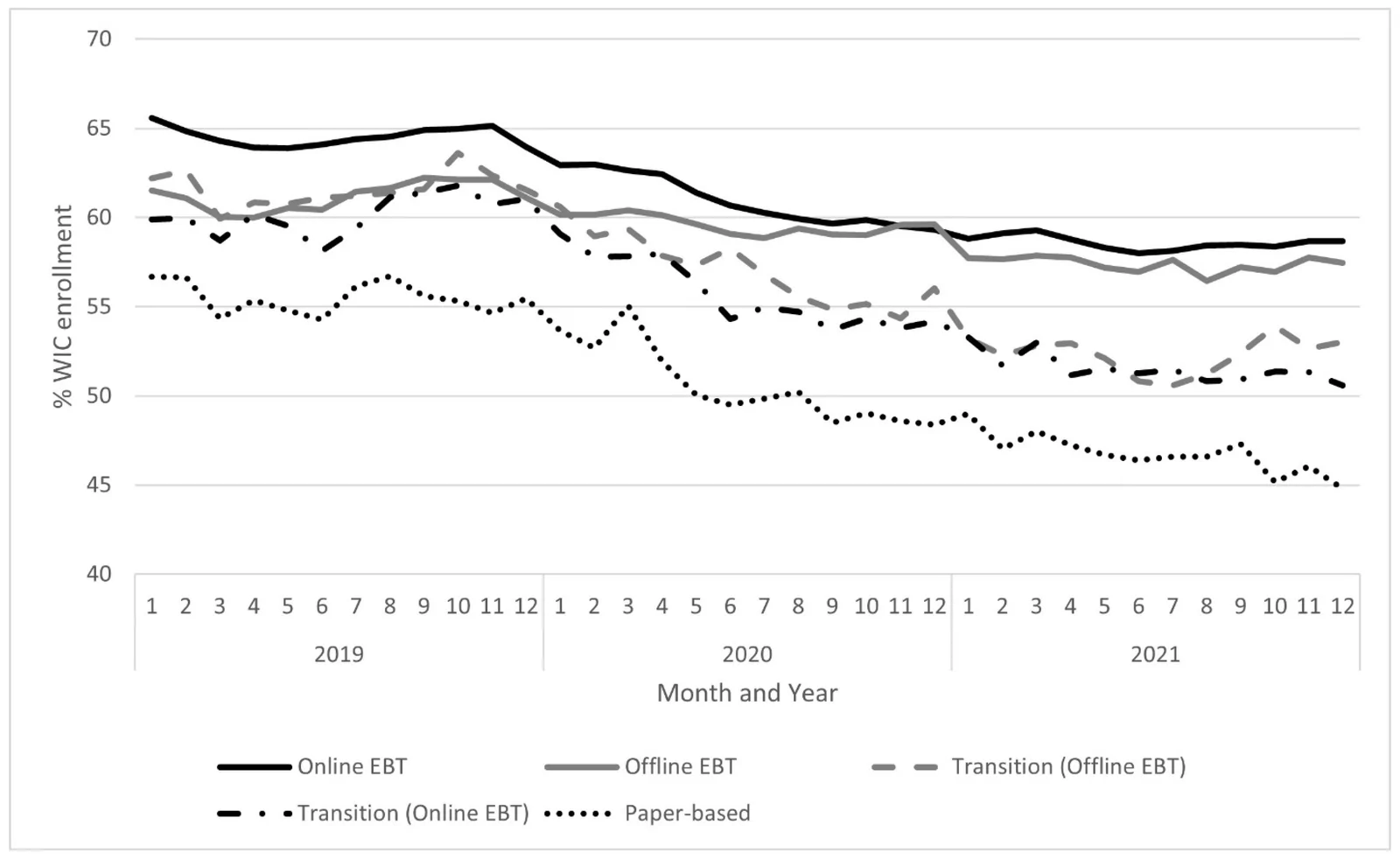
Trends in prenatal WIC participation by state benefit issuance group, U.S., 2019–2021.

Unadjusted monthly trends in prenatal WIC participation were examined over the study period by state benefit issuance group (Figure 2, Supplemental Table S1). Overall, online EBT states had the highest rates of WIC participation prior to the pandemic but declined between 2020 and 2021—with WIC participation differences in online and offline narrowing by the end of 2021 (58.7% for online and 57.5% for offline EBT by December 2021). States that were transitioning to an EBT system during the pandemic had prenatal WIC participation percentages comparable to offline EBT states prior to the pandemic (~61% for all 3 groups by December 2019) but declined more rapidly during the pandemic (57.7% for offline EBT compared to 53.0 and 50.6 for states transitioning to offline and online, respectively, by December 2021). Finally, states that remained on paper vouchers had the lowest levels of prenatal WIC participation, which also declined more rapidly during the pandemic (44.8% by December 2021)

In LPMs, the percentage of births in which the mother enrolled in WIC during her pregnancy was examined by state benefit issuance group (online, offline, transition, and paper-based) across the three time periods: pre-pandemic, pandemic 2020, and pandemic 2021 (Table 2). Patterns between unadjusted and adjusted analyses were similar. After adjustment, the offline EBT states (β = −5.66, 95% CI: −9.75, −1.58), transition states (β = −3.49, 95% CI: −8.69, 1.71), and paper-based states (β = −9.69, 95% CI: −14.97, −4.40) had lower prenatal WIC participation compared with online EBT states prior to the pandemic. States that had online EBT systems experienced significant declines in WIC participation in both pandemic periods compared to before the pandemic (2020: β = −4.61, 95% CI: −5.86, −3.35; 2021: β = −6.09, 95% CI: −7.79, −4.38). These trends over time were not statistically significantly different for states with offline EBT systems (*p*-value for interaction = 0.08 and 0.30 for change in offline compared to online in 2020 and 2021, respectively). In comparison, states that were in the process of transitioning to an EBT system (β = −1.00, 95% CI: −1.94, −0.07 in 2020; β = −2.76, 95% CI: −4.61, −0.91 in 2021) or remained on paper-based systems throughout the pandemic (β = −1.98, 95% CI: −3.59, −0.37 in 2020; β = −3.17, 95% CI: −5.47, −0.87 in 2021) continued to decline significantly during the pandemic relative to states with online EBT systems.

**Figure 3:**
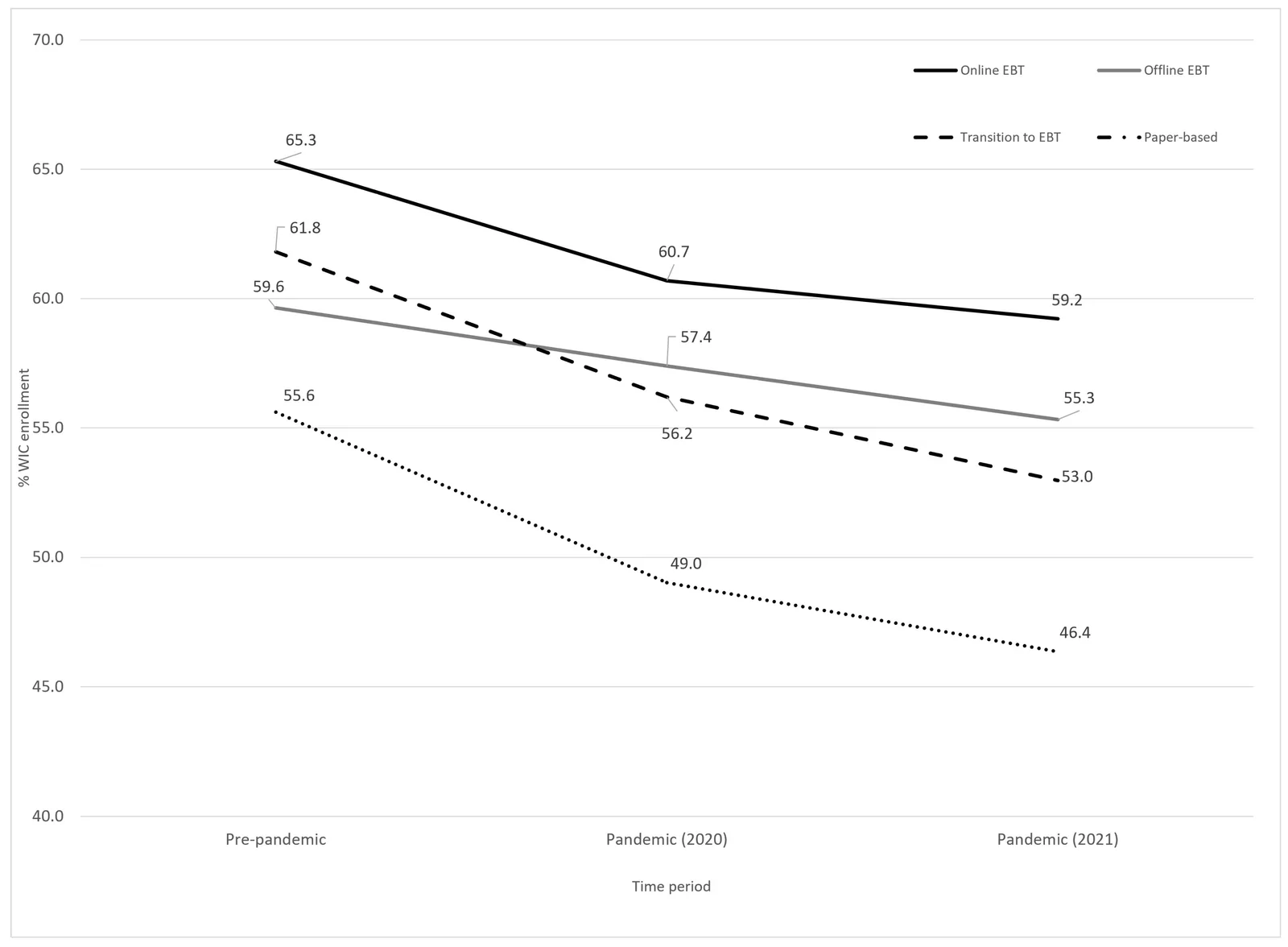
Adjusted percentages of prenatal WIC participation by state benefit issuance system and time period.

Adjusted probabilities in WIC participation were plotted by time period and benefit issuance group (Figure 3, Supplemental Table S2) and showed the largest absolute declines in WIC participation for states on paper-based systems (9.2 percentage point decline), followed by states transitioning to EBT systems (8.8 percentage point decline), online EBT systems (6.1 percentage point decline), and offline EBT systems (4.3 percentage point decline)—widening the gap between states with modernized benefit issuance systems and those without.

## 4. Discussion

Among over 4.5 million U.S. births examined in this study, state and individual maternal and infant demographic and health characteristics changed in relatively comparable ways for each WIC benefit issuance group between 2019 and 2021. Prior to the start of the pandemic, states with online EBT systems had higher prenatal WIC enrollment compared with states using offline EBT systems, states transitioning to EBT systems, and those still using paper vouchers throughout the study period (2019–2021). Despite the USDA administering waivers to state WIC agencies to minimize pandemic-related disruption to services, both unadjusted and adjusted prenatal WIC participation declined during the COVID-19 pandemic for all benefit issuance groups. The extent of these declines was more pronounced among states transitioning to an EBT system and states that remained on paper-based systems compared with states that had EBT systems in place prior to March 2020. We found minimal difference, both statistically and descriptively, in prenatal WIC participation between online vs. offline EBT systems, suggesting declines were attenuated during the pandemic in states with either type of EBT system.

WIC enrollment during pregnancy has been found to support positive outcomes for both mother and child [2]. However, the pandemic brought unique constraints for this population. Our findings show that prenatal WIC participation declined across all benefit issuance systems, consistent with USDA reports of declines in WIC participation among pregnant and postpartum women during this same time period [26], and that these declines were more pronounced earlier in the pandemic (2020) compared to 2021. Prior studies found that pregnant women were more vulnerable to developing severe outcomes from COVID-19 and less likely to receive SARS-CoV2 vaccination, which may have also deterred engagement with WIC programs if they were not aware of remote services or other social distancing precautions (e.g., benefit issuance outside WIC clinics) during this time [35,36,37].

Health departments were also grappling with greater resource and service constraints in the early phase of the COVID-19 pandemic [19], which may have affected WIC agency adaptations to remote certifications and eligibility verifications. Qualitative studies of mothers and families navigating food assistance programs during the pandemic identify both fears about accessing assistance as well as experiencing delays in accessing benefits [38], suggesting both individual and system-level interactions influenced engagement. Another study of California WIC participants and agencies (online EBT) showed high participant satisfaction with remote service delivery but also noted potential challenges in verifying certain eligibility criteria (e.g., height/weight or bloodwork) and postal delays in issuing new WIC cards [39].

Although these factors may explain initial declines in prenatal WIC participation, we found significant variation based on benefit issuance systems over time. Specifically, states with paper-based systems or states that were transitioning from paper to EBT systems during the pandemic showed larger and continuing declines in WIC participation across both 2020 and 2021. During the pandemic, WIC agencies in non-EBT states relied mainly on mail to reissue benefits, while the majority of WIC agencies in online EBT states relied on remote benefit issuance (92%), and offline EBT states relied on pick-up inside or outside the WIC clinic (71%) [19]. Studies conducted prior to the pandemic report significant barriers to using paper vouchers, including a diminished retail experience, greater time costs related to accessing and using benefits, and increased stigma during purchasing [12,13,14,40]. These barriers to utilization may have been further compounded during the pandemic due to both WIC agency program constraints and individual engagement with these systems. Additionally, states transitioning from paper-based systems may have stalled efforts due to other priorities and resource constraints. In comparison, pandemic-related declines were mitigated in states that had any EBT system in place prior to the start of the pandemic compared to states that did not. This finding is consistent with an earlier study prior to 2020 showing increased WIC participation with state adoption of either online or offline EBT systems compared with paper vouchers [41]. However, it contrasts with two other studies examining WIC participation during the COVID-19 pandemic, which found greater increases in WIC participation among states with online (vs. offline) EBT systems [16,27]. This discrepancy may reflect differences in study populations and how WIC participation was measured across studies. First, we conditioned our sample on WIC-eligibility (i.e., births paid for by Medicaid) to ensure enrollment trends were independent of changes in eligibility status during the COVID-19 pandemic. We also focused on WIC enrollment during pregnancy, which may have had differing pandemic-related effects. Our measure of self-reported WIC participation during pregnancy is likely to reflect new WIC enrollments, whereas prior studies used USDA WIC monthly benefit summary reports that encompass both new and ongoing WIC participation. As such, online EBT systems may have higher mechanical retention generated through automatic benefit issuance. These contrasting results underscore the importance of population-specific analyses and the use of multiple measures of WIC enrollment to inform programmatic responses to public health emergencies or other program-related shocks. Further studies are needed to distinguish the effects of WIC policies and benefit issuance systems on enrollment, retention, and redemption of benefits.

There are limitations to consider when interpreting findings from our study. First, although we adjusted for several state-specific covariates, including average household poverty, unemployment, and income levels, we did not adjust for COVID-19-specific responses or other state policies that may have been related to a state’s health system capacity and their adoption of a specific benefit issuance system prior to the pandemic. It is also plausible that several of these factors do not operate as confounders but rather as a context by which certain benefit issuance systems may have led to staff being better able to process new certifications and more easily issue food package benefits during a time of increased health department demand. Second, birth certificate data does not include household income or other eligibility requirements for WIC enrollment. Instead, we used births paid for by Medicaid at the time of delivery as a proxy for WIC eligibility in this study. Thus, we may have excluded some WIC-eligible pregnant women in our analyses. Additionally, Medicaid status at delivery may not have reflected Medicaid status earlier in pregnancy or prior to WIC enrollment; however, we do not anticipate a large change in household income during pregnancy for most individuals who were eligible for Medicaid at delivery. Lastly, medical and health information on the birth certificate has been shown to be underreported for some items, such as diabetes, hypertension, and cigarette use during pregnancy. Therefore, we cannot rule out the possibility that the estimates in our analysis are influenced by unmeasured or residual confounding [42].

## 5. Conclusions

Our findings, in combination with other research, have broader implications for understanding how WIC benefit issuance systems may mitigate service disruptions and enhance WIC enrollment during public health emergencies or system-level shocks. The COVID-19 pandemic resulted in office closures, staffing constraints, and other supply disruptions that may have exacerbated existing administrative burdens related to WIC enrollment [19]. These burdens are commonly conceptualized as learning costs related to program knowledge and eligibility, compliance costs associated with enrollment and retention, and psychological costs due to related stress and stigma of participation [43]. Using this framework, states could tailor outreach and communication of program disruptions and responses to specific subgroups, such as pregnant women, to ensure timely access to WIC benefits. To address compliance costs, programs may partner with other health systems that engage regularly with pregnant populations or enhance multi-channel enrollment options during periods of disruption (e.g., in person, online, phone, or mail delivery) [44]. Other studies have shown that EBT cards alleviated stigma and enhanced purchasing efficiency, which can further minimize the psychological burden of participation [12,13,14]. This study could be used to inform planning for potential service-delivery impacts resulting from future shocks, such as weather-related events, abrupt policy shifts, or other supply chain disruptions (e.g., the 2022 U.S. infant formula disruption) [45,46].

Currently, all states have transitioned to an EBT system; however, continued modernization of service delivery efforts is underway, and new modes of benefit issuance are being considered. A proposed rule issued by the Food and Nutrition Administration in February 2023 would require all states to transition to online EBT systems. Additionally, in February 2025, the U.S. Congress introduced the MODERN WIC Act of 2025, which would make permanent states abilities to use remote certification and remote benefit issuance options [47]. If enacted, the Food and Nutrition Administration would be required to report to Congress on the impacts of remote service provision options for certifications and participant satisfaction. This research underscores that attention to heterogenous effects for different participant groups or use of different measures in evaluation of remote service provision use may inform Congress and state WIC agencies on effective and responsive strategies to optimize WIC enrollment and benefit use to meet the nutritional needs of low-income women and families.

## Figures and Tables

**Table 1:** Percent distribution of sociodemographic, maternal, infant health, and state characteristics by state WIC agency benefit issuance system at the start of the COVID-19 pandemic among births paid for by Medicaid, 2019–2021.

	Online EBT Systems			Offline EBT Systems			Transitioning to EBT System			Paper-Based System		
	**2019**	**2020**	**2021**	**2019**	**2020**	**2021**	**2019**	**2020**	**2021**	**2019**	**2020**	**2021**
**Total births**	989,874	945,595	936,983	353,893	344,517	338,691	131,344	127,323	124,026	92,620	89,454	90,385
	Sociodemographic characteristics											
Age (years)												
<20	7.89	7.56	7.22	10.32	9.92	9.37	9.22	8.92	8.3	8.41	8.14	7.47
20–24	26.92	26.52	26.02	30.7	30.22	29.98	29.53	29.02	28.58	27.48	27.21	26.55
25–29	30.6	30.17	29.89	30.2	30.05	29.88	30.56	30.45	30.13	30.95	30.41	30.01
30–34	21.25	21.96	22.67	18.52	19.14	19.75	19.29	19.99	20.81	20.61	21.01	22.21
35–39	10.63	10.95	11.28	8.34	8.64	8.88	9.24	9.25	9.74	10.07	10.55	10.87
40+	2.71	2.85	2.93	1.91	2.04	2.14	2.16	2.37	2.45	2.48	2.69	2.89
Marital Status ^1^												
Married	34.35	33.55	33.52	31.86	31.86	31.9	31.28	30.29	30.39	32.25	31.17	31.54
Unmarried	65.65	66.45	66.48	68.14	68.14	68.1	68.72	69.71	69.61	67.75	68.83	68.46
Highest education level obtained												
Less than high school	22.56	21.88	20.88	23.19	22.07	20.83	20.57	20.12	19.25	18.99	18.39	17.54
High school	39.76	40.47	40.94	42.24	43.4	44.07	39.11	39.87	40.48	44.38	45.5	45.34
Some college	22.69	22.33	22.03	22.58	22.06	21.72	24.98	24.46	23.9	19.92	18.95	18.82
College or more	14.99	15.32	16.16	11.99	12.46	13.38	15.34	15.55	16.36	16.72	17.16	18.3
Race and ethnicity												
Non-Hispanic White	36.96	37.00	36.65	31.68	31.34	31.14	41.53	41.36	41.73	29.51	28.77	28.95
Non-Hispanic Black	20.2	20.23	19.82	22.49	22.28	21.8	27.54	27.57	26.90	41.74	41.58	40.04
Hispanic	33.13	33.27	34.18	40.32	40.95	41.61	21.22	21.59	21.92	22.92	23.74	25.16
Non-Hispanic Asian	4.28	3.94	3.76	2.07	1.98	1.95	3.01	2.92	2.84	2.81	2.80	2.60
Non-Hispanic NHOPI	0.33	0.34	0.34	0.19	0.18	0.2	1.23	1.23	1.15	0.06	0.07	0.07
Non-Hispanic AIAN	1.45	1.45	1.43	0.84	0.81	0.75	0.89	0.90	0.86	0.09	0.09	0.09
Non-Hispanic Multiracial/Unknown	3.65	3.76	3.82	2.41	2.46	2.54	4.58	4.43	4.59	2.87	2.96	3.09
	Maternal Health											
Parity												
1	32.2	32.27	32.06	32.5	32.94	32.77	31.78	32.24	31.84	33.67	34.28	33.45
2	29.24	29.20	29.28	28.95	28.56	28.84	28.85	28.59	28.68	29.14	28.86	28.97
3	19.79	19.57	19.54	20.03	19.95	19.73	19.82	19.76	19.81	19.28	18.85	19.26
4+	18.76	18.96	19.12	18.51	18.55	18.66	19.55	19.42	19.67	17.92	18.02	18.32
Body Mass Index												
Underweight < 18.5	3.64	3.41	3.30	3.68	3.45	3.32	3.63	3.48	3.33	3.64	3.39	3.16
Normal 18.5–24.9	35.99	34.9	33.92	35.15	33.79	32.75	34.67	33.69	32.54	36.06	34.92	33.86
Overweight 25.0–29.9	27.16	27.31	27.49	26.76	27	27.23	25.89	26.05	26.09	26.64	26.97	27.29
Obese 30.0+	33.21	34.39	35.29	34.41	35.76	36.71	35.81	36.78	38.05	33.66	34.72	35.7
Smoking status												
None	86.6	87.38	89.22	86.60	87.38	89.22	87.47	88.45	90.18	83.88	84.88	86.55
3 months prior to pregnancy only	2.45	2.28	1.95	2.45	2.28	1.95	2.65	2.32	1.99	2.60	2.41	2.03
During pregnancy and 3 months prior	10.95	10.33	8.83	10.95	10.33	8.83	9.89	9.23	7.83	13.52	12.71	11.42
Diabetes												
None	91.72	90.69	90.12	92.9	91.84	91.23	92.13	90.99	90.47	92.99	91.92	91.24
Pre-pregnancy	1.19	1.28	1.31	1.14	1.23	1.29	1.16	1.19	1.3	1.14	1.27	1.27
Gestational	7.09	8.03	8.57	5.95	6.93	7.48	6.71	7.82	8.23	5.87	6.81	7.49
Hypertension												
None	90.13	89.09	88.36	88.90	88.24	87.39	88.28	87.64	86.19	89.97	88.50	87.97
Pre-pregnancy	2.33	2.64	2.79	2.43	2.75	2.96	3.01	3.29	3.65	2.75	3.24	3.26
Gestational	7.54	8.27	8.85	8.67	9.01	9.65	8.71	9.07	10.16	7.28	8.26	8.78
Prenatal Care Initiation												
1st trimester	70.28	70.48	71.22	64.01	64.43	65.95	67.43	68.25	69.79	64.95	66.07	68.08
2nd trimester	21.44	21.55	20.42	24.89	24.72	23.05	23.47	23.24	21.67	24.55	23.56	22.45
3rd trimester or none	8.28	7.98	8.37	11.10	10.84	11.01	9.09	8.51	8.53	10.49	10.37	9.47
Birth outcomes												
Preterm Birth (%)	10.98	11.13	11.49	12.29	12.11	12.62	12.49	12.39	12.78	12.16	12.18	12.22
Low birthweight (%)	9.46	9.57	9.83	10.2	10.12	10.47	10.8	10.63	10.95	11.09	11.03	11.67
	State Characteristics ^2^											
Poverty rate, mean (SD)	12.24 (1.78)	12.79 (1.88)	12.63 (1.75)	14.12 (2.04)	14.49 (1.86)	14.58 (2.09)	12.81 (3.27)	13.06 (3.13)	13.06 (3.02)	11.99 (1.92)	12.93 (2.18)	12.85 (1.92)
Monthly unemployment rate, mean (SD)	3.64 (0.58)	8.19 (4.09)	5.33 (1.58)	3.90 (0.50)	7.97 (2.92)	5.62 (0.82)	3.76 (0.96)	7.88 (3.93)	5.24 (1.21)	3.62 (0.42)	7.44 (3.54)	4.83 (1.33)
Median HH income (per 1k), mean (SD)	68.21 (10.68)	66.63 (10.44)	71.49 (10.94)	60.69 (5.11)	60.52 (5.11)	63.91 (5.65)	63.50 (10.30)	63.19 (9.712)	66.87 (10.03)	71.02 (11.93)	69.99 (11.88)	74.96 (11.01)

^1^ Excludes California. Beginning in 2017, NCHS cannot release record-level data on the marital status of the mother for births occurring in or to residents of California due to state statutory restrictions. NOTE: ^2^ State characteristics are aggregates from each state that are all averaged to get the mean per year.

**Table 2:** Differences in the percentage of prenatal WIC participation by EBT status and timing of birth among births paid for by Medicaid, 2019–2021.

	Unadjusted Model	Adjusted Model ^A^
Total Births	4,509,595	4,242,881
Timing of Birth (Online EBT Group)		
Pre-Pandemic	Ref	Ref
Pandemic 2020	−4.04 (−4.71, −3.37) *	−4.61 (−5.86, −3.35) *
Pandemic 2021	−5.53 (−6.23, −4.83) *	−6.09 (−7.79, −4.38) *
Benefit Issuance Group (Pre-pandemic period)		
Online	Ref	Ref
Offline	−3.13 (−7.61, 1.35)	−5.66 (−9.75, −1.58) *
Transition	−4.02 (−10.36, −2.31)	−3.49 (−8.69, 1.71)
Paper-Based	−9.12 (−16.13, −2.10) *	−9.69 (−14.97, −4.40) *
Interaction (Benefit Issuance Group × Timing of Birth)		
Offline × Pandemic 2020	2.32 (−0.50, 5.15)	2.37 (−0.31, 5.05)
Offline × Pandemic 2021	1.92 (−1.56, 5.40)	1.78 (−1.61, 5.16)
Transition × Pandemic 2020	−1.07 (−1.90, −0.25) *	−1.00 (−1.94, −0.07) *
Transition × Pandemic 2021	−2.81 (−4.80, −0.82) *	−2.76 (−4.61, −0.91) *
Paper-Based × Pandemic 2020	−1.69 (−3.34, −0.04) *	−1.98 (−3.59, −0.37) *
Paper-Based × Pandemic 2021	−2.75 (−5.21, −0.28) *	−3.17 (−5.47, −0.87) *

NOTE: All coefficients and confidence intervals are multiplied by 100 to represent change in %. The confidence intervals are reported after the coefficients. * *p* < 0.05; ^A^ demographic-adjusted model for maternal age, education, race & ethnicity, BMI, smoking, hypertension, diabetes, prenatal care initiation trimester, and state characteristics (poverty level, unemployment rate, and median household income).

## Data Availability

Study data can be requested from the National Vital Statistics System at the National Center for Health Statistics through a restricted-use data request application. Access is subject to agency approval and applicable data use and confidentiality requirements.

## Supplementary Materials

The following supporting information can be downloaded at: https://www.mdpi.com/article/10.3390/nu18213484/s1, Table SI: Monthly prenatal WIC enrollment percentages by state benefit issuance group, United States, 2019–2021. Table S2: Adjusted percentages of prenatal WIC enrollment by state benefit issuance system and time period.
