# Supplementary material for: State Benefit Issuance Systems and Prenatal WIC Participation Trends During the COVID-19 Pandemic
Source: Nutrients. 2026 Oct 23;18(21):3484. doi: 10.3390/nu18213484 (PMC13764909; doi:10.3390/nu18213484)
Supplement: Supplementary file 1 [file nutrients-18-03484-s001.zip › nutrients-4532205-supplementary.pdf]

Supplemental Table S1. Monthly prenatal WIC enrollment percentages by state benefit issuance group, United States, 2019-2021

Year	Month	State benefit issuance group				
		Online EBT	Offline EBT	Transition (Offline EBT)	Transition (Online EBT)	Paper-based
2019	1	65.57	61.52	62.21	59.89	56.67
	2	64.84	61.09	62.64	59.95	56.65
	3	64.31	60.04	59.94	58.71	54.36
	4	63.91	60.01	60.85	60.18	55.36
	5	63.89	60.55	60.78	59.53	54.77
	6	64.08	60.45	61.11	58.15	54.27
	7	64.38	61.46	61.2	59.4	56.15
	8	64.52	61.65	61.4	61.14	56.7
	9	64.92	62.24	61.57	61.39	55.6
	10	64.96	62.13	63.63	61.79	55.32
	11	65.13	62.13	62.38	60.73	54.65
	12	63.94	61.11	61.55	61.06	55.46
2020	1	62.93	60.16	60.59	59.09	53.65
	2	62.97	60.15	58.93	57.81	52.7
	3	62.64	60.4	59.35	57.83	55.04
	4	62.42	60.14	57.85	57.97	51.95
	5	61.39	59.64	57.33	56.37	50.04
	6	60.68	59.07	58.28	54.31	49.52
	7	60.28	58.83	56.81	54.96	49.84
	8	59.94	59.4	55.57	54.71	50.22
	9	59.66	59.05	54.84	53.7	48.49
	10	59.85	59.03	55.14	54.35	49.02
	11	59.53	59.6	54.33	53.79	48.59
	12	59.32	59.63	56.04	54.17	48.4
2021	1	58.82	57.73	53.21	53.28	49.01
	2	59.12	57.67	52.27	51.7	47.04
	3	59.27	57.88	52.82	52.98	48
	4	58.77	57.76	52.97	51.15	47.25
	5	58.32	57.2	52.11	51.52	46.71
	6	58.01	56.94	50.82	51.28	46.38
	7	58.15	57.63	50.58	51.45	46.59
	8	58.45	56.44	51.22	50.84	46.58
	9	58.48	57.22	52.39	50.92	47.32
	10	58.38	56.96	53.89	51.35	45.18
	11	58.68	57.77	52.64	51.34	46.04
	12	58.67	57.47	53.02	50.6	44.8

Supplemental Table S2. Adjusted percentages of prenatal WIC enrollment by state benefit issuance system and time period

	Pre-pandemic (2019 – March 2020)	Pandemic (April 2020-Dec. 2020)	Pandemic (Jan. 2021-Dec. 2021)
**Online EBT**	65.3 (61.9, 68.7)	60.7 (57.3, 64.0)	59.2 (55.9, 62.6)
**Offline EBT**	59.6 (57.7,61.6)	57.4 (54.4, 60.4)	55.3 (51.8, 58.8)
**Transition to EBT**	61.8 (57.5, 66.2)	56.2 (51.8, 60.6)	53.0 (49.9, 56.1)
**Paper voucher**	55.6 (51.4, 59.8)	49.0 (46.0, 52.0)	46.4 (43.3, 49.3)
